# Vitamin D and Obesity: Two Interacting Players in the Field of Infertility

**DOI:** 10.3390/nu11071455

**Published:** 2019-06-27

**Authors:** Julia K. Bosdou, Eirini Konstantinidou, Panagiotis Anagnostis, Efstratios M. Kolibianakis, Dimitrios G. Goulis

**Affiliations:** 1 Unit for Human Reproduction, 1st Department of Obstetrics and Gynaecology, Aristotle University of Thessaloniki, 541 24 Thessaloniki, Greece; 2 Unit of Reproductive Endocrinology, 1st Department of Obstetrics and Gynaecology, Aristotle University of Thessaloniki, 541 24 Thessaloniki, Greece

**Keywords:** vitamin D, obesity, infertility, assisted reproductive technologies

## Abstract

Obesity plays an important role in human fertility in both genders. The same is true for vitamin D, for which accumulating evidence from observational human studies suggests a key role for both male and female fertility. In the latter case, however, robust data from relevant interventional studies are currently lacking. It is also not clear whether obesity and vitamin D deficiency, besides their independent effect on human infertility, act in synergy. Several pathogenetic mechanisms may be proposed as a linkage between vitamin D deficiency and obesity, with respect to infertility. In any case, the independent contribution of vitamin D deficiency in obese infertile states needs to be proven in interventional studies focusing on either vitamin D supplementation in obese or weight loss strategies in vitamin D-deficient infertile patients.

## 1. Introduction

Vitamin D plays a crucial role in calcium and phosphate homeostasis, by increasing intestinal calcium absorption and renal calcium reabsorption [1]. It is found in two major forms, D_2_ (ergocalciferol) and D_3_ (cholecalciferol). The former is produced by ergosterol upon irradiation in plants and fungi. The latter is produced by 7-dehydrocholesterol upon irradiation in the epidermis [2]. After hydroxylation at carbon 25 [producing 25-hydroxyvitamin D, 25(OH)D], it is transported to the kidney, where it is hydroxylated by 1α-hydroxylase (CYP27B1) at the carbon 1 of the A ring, producing 1,25-dihydroxy-vitamin D [1,25(OH)_2_D], the active form of vitamin D [3]. CYP27B1 is also present in extrarenal sites, such as macrophages, osteoblasts, epithelial, endocrine, placental and cancer cells [4]. The mechanism of 1,25(OH)_2_D action involves its binding to vitamin D receptor (VDR), a transcription factor, member of the steroid hormone nuclear receptor family [2]. VDR and CYP27B1 are expressed in various cells, indicating that vitamin D is characterized by a plethora of extra-skeletal actions, such as those on the immune and cardiovascular system [5].

Vitamin D deficiency is defined as 25(OH)D concentrations <20 ng/mL (50 nmol/L), whereas vitamin D insufficiency as 25(OH)D concentrations 20–30 ng/mL (50–75 nmol/L) [6]. The prevalence of vitamin D deficiency ranges from 8 to 90% in Europe (reaching >50% in Western European populations) and from 14 to 89% in North America [7]. Indeed, vitamin D deficiency has been associated with an increased risk of various autoimmune diseases (such as multiple sclerosis, rheumatoid arthritis and type 1 diabetes mellitus), susceptibility to infections [8], as well as type 2 diabetes mellitus (T2DM), metabolic syndrome and cardiovascular disease (CVD) [9]. A key role in human infertility has also emerged recently for vitamin D. This is mainly attributed to the presence of both VDR and CYP27B1 in various tissues of the reproductive system in both sexes [10].

On the other hand, obesity, defined as body mass index (BMI) > 30 kg/m^2^ [11], is also associated with severe adverse health consequences, depending on the amount of adiposity and its distribution within the body [12]. Besides the well-recognized health concerns, such as T2DM, CVD and cancer, there is also a strong link between obesity and infertility [13,14]. Many pathogenetic mechanisms for this interplay have been proposed. With regard to male infertility, these include the aromatization of testosterone to estrogen in peripheral tissues, decreased sex hormone-binding globulin (SHBG) production in the liver [15,16], increased endorphin concentrations [leading to lower luteinizing hormone (LH) pulse and gonadotropin releasing hormone (GnRH) production] [17] and increased oxidative stress, which promotes sperm DNA damage [18]. With respect to female infertility, the underlying mechanisms include functional alterations of the hypothalamic–pituitary–gonadal (HPG) axis due to insulin resistance and relative hyperandrogenaemia [14], disordered secretion of gonadotrophins and hyperleptinaemia, leading to impaired folliculogenesis and ovulatory dysfunction [19,20,21,22].

The purpose of this review is to provide up-to-date evidence regarding the impact of vitamin D deficiency and obesity on male and female infertility. Additionally, a basis for an interaction between obesity and vitamin D status is proposed, exploring at the same time gaps in knowledge and deficiencies in published research.

## 2. Vitamin D and Infertility

### 2.1. Vitamin D and Male Infertility (Observational Studies)

An accumulative body of evidence from observational studies suggests a potentially key role for vitamin D in male reproductive function, including semen quality and androgen status. This is indicated by a positive correlation between vitamin D concentrations and sperm motility [23,24,25] and vitamin D concentrations and normal sperm morphology in infertile men [26]. Particularly, a cross-sectional study including 300 men showed that men with severe vitamin D deficiency [25(OH)D <10 ng/mL] had a lower proportion of motile spermatozoa (62% vs. 70%; *p* = 0.027), progressive motile spermatozoa (56% vs. 64%; *p* = 0.035) and % of morphologically normal spermatozoa (6% vs. 8%; *p* = 0.044) compared with those with vitamin D sufficiency [26]. Similar results were obtained from a subsequent prospective study including 1427 infertile men, which demonstrated higher sperm motility in men with 25(OH)D >30 ng/mL compared with those with 25(OH)D <10 ng/mL [45% (31–63%) vs. 34% (22–54%), respectively; *p* = 0.030]. However, no differences were observed regarding total sperm count, sperm concentration, sperm volume or sperm morphology [27]. On the other hand, a cross-sectional study, including 170 men, showed a U-shaped correlation of vitamin D concentrations with semen parameters, supporting that not only low, but also high vitamin D concentrations are associated with impaired sperm quality [28]. In detail, men with high vitamin D concentrations (≥50 ng had lower sperm concentration [46.7 (95% confidence interval (CI) 27.2 to 73.9) vs. 84.0 (95% CI 70.3 to 99.3) million/mL; *p* < 0.05), progressive motile sperm [38.4% (95% CI 29.3 to 49.2) vs. 52.6% (95% CI 47.6 to 58.0); *p* < 0.05] and normal sperm morphology [18% (95% CI 12.1 to 25.6) vs. 27.4% (95% CI 23.8 to 31.3); *p* < 0.05) compared with those with 25(OH)D concentrations between 20 and 50 ng/mL [28].

Furthermore, serum 25(OH)D concentrations are associated not only with semen quality, but also with androgen status. Data from cross-sectional studies have shown a positive association between 25(OH)D and testosterone concentrations [29,30,31]. In particular, a large cross-sectional study of 2299 men demonstrated higher total testosterone concentrations, free androgen index, and lower SHBG concentrations in vitamin D-sufficient compared with vitamin D-insufficient or -deficient men (*p* < 0.05 for all) [30]. Likewise, results from another cross-sectional survey of 3369 men in eight European centers (the European Male Ageing Study) supported a linkage between vitamin D deficiency and secondary or compensated hypogonadism [relative risk ratio (RRR) = 1.16, *p* = 0.05], as 25(OH)D concentrations were positively associated with total and free testosterone and negatively with estradiol and LH concentrations [31].

### 2.2. Vitamin D and Male Infertility (Interventional Studies)

Few interventional studies have assessed the effect of vitamin D supplementation on semen quality, male fertility and testosterone concentrations. In a recent triple-blinded, randomized clinical trial, 330 men with infertility and vitamin D insufficiency received either a single dose of 300,000 IU cholecalciferol (followed by 1400 IU/day, combined with calcium 500 mg/day for 150 days) or placebo. Although there was no difference in sperm concentration, the number of spontaneous pregnancies was higher in the vitamin D compared with the placebo group (7.3% vs. 2.4%; 95% CI −0.6% to +10.5%) [32]. In another study, 86 infertile men with idiopathic oligoasthenospermia were randomized to oral cholecalciferol 200 IU/day with calcium 600 mg/day, or a combination of vitamin E 100 mg plus vitamin C 100 mg, t.i.d. After three months, semen quality, especially the progressively motile sperm count per ejaculate and the proportion of progressively motile sperm were increased only in the vitamin D group. In particular, the mean count of progressively motile sperm per ejaculate was increased from 9.8 ± 3.7 × 10^6^ to 21.5 ± 6.5 × 10^6^ (*p* < 0.05) in the vitamin D group, while it was increased from 9.5 ± 6.3 × 10^6^ to 12.4 ± 4.4 × 10^6^ (*p* > 0.05) in the control group. The proportion of progressively motile sperm was also increased, from 18.4 ± 9.8% to 28.3 ± 4.5% (*p* < 0.05) in the vitamin D group, while it did not increase in the control group (17.8 ± 5.3% to 21.4 ± 2.4%; *p* > 0.05). In addition, pregnancy rates were higher in the vitamin D group (16.3%) compared with the control group (2.3%) (*p* < 0.05) [33].

Additional interventional studies assessed the association between vitamin D and androgen status. A randomized controlled trial (RCT) in vitamin D-deficient men evaluated the effect of vitamin D supplementation (cholecalciferol 3330 IU/day, *n* = 31) on testosterone concentrations, compared with placebo (*n* = 23) [34]. An increase in total (10.7 ± 3.9 to 13.4 ± 4.7 nmol/L; *p* < 0.001), bioactive (from 5.2 ± 1.9 to 6.3 ± 2.0 nmol/L; *p* < 0.001) and free testosterone concentrations (from 0.22 ± 0.08 nmol/L to 0.27 ± 0.09 nmol/L; *p* < 0.001) was observed in the vitamin D supplemented group, while there was no change in the placebo group [34]. Similar results were demonstrated by a prospective study, including 102 men who received a single dose of ergocalciferol (600,000 IU). Significant increase in serum total testosterone concentrations (from 12.46 ± 3.30 to 15.99 ± 1.84 nmol/L, *p* < 0.01) and erectile function scores (from 13.88 ± 3.96 to 20.25 ± 3.24, *p* < 0.01) were observed after 12 months [35].

The aforementioned data suggest a potentially adverse effect of low vitamin D status on male fertility, although a U-shape is more representative of its association with infertility. Vitamin D supplementation may improve sperm quality and increase spontaneous pregnancy rates and testosterone concentrations. Thus, while there is no level 1 evidence, vitamin D supplementation, achieving sufficient but not high (i.e., 30–50 ng/mL) 25(OH)D concentrations, may have a beneficial effect on male infertility [36].

### 2.3. Vitamin D and Female Infertility (Observational Studies)

Recently, research has focused on the role of vitamin D concentrations in women undergoing assisted reproductive technologies (ART). Based on data reported in a systematic review and meta-analysis of 11 cohort studies, including 2700 women, investigating the association between vitamin D status and ART outcome, higher live birth rates have been reported in vitamin D-sufficient compared with vitamin D-deficient and -insufficient women [odds ratio (OR): 1.33 (95% CI 1.08 to 1.65), seven studies] [37]. Similar results were shown in another recently published meta-analysis of nine cohort studies, which supported the decreased live birth rates after in vitro fertilization (IVF)/intracytoplasmic sperm injection (ICSI) in women with vitamin D deficiency compared with those of sufficient vitamin D status [relative risk (RR): 0.74 (95% CI 0.58 to 0.90), three studies] [38].

Vitamin D has also been involved in the development of specific gynecological conditions affecting fertility, such as endometriosis and polycystic ovarian syndrome (PCOS). In particular, a cohort study of 49 women showed a significant linear correlation between 25(OH)D concentrations and the diameter of ovarian endometriomas (r = −0.3, *p* = 0.03) [39]. Moreover, a prospective comparative study, evaluating 25(OH)D concentrations in 135 women with endometriosis and 90 controls, showed that the incidence of women with vitamin D deficiency/insufficiency was significantly higher in women with endometriosis compared with the control group (80% vs. 33.3%; *p* < 0.001) [40]. Moreover, the impact of vitamin D concentrations on reproductive outcomes, in women with PCOS undergoing ovulation induction, has been investigated in a retrospective cohort study (*n* = 540). This study showed that vitamin D-deficient women were less likely to achieve ovulation compared with those with 25(OH)D >20 ng/mL (OR 0.43, 95% CI 0.25 to 0.76, *p* = 0.006). Furthermore, live birth rates after ovulation induction were increased by 2% for each 1 ng/mL increase in 25(OH)D concentrations (OR 1.02, 95% CI 1.00 to 1.04, *p* = 0.040) [41]. However, data are still insufficient in order to establish a possible causality between vitamin D and endometriosis or vitamin D and PCOS. Further studies are needed to confirm these associations.

### 2.4. Vitamin D and Female Infertility (Interventional Studies)

Few RCTs currently exist evaluating the effect of vitamin D supplementation on ART outcome, yielding inconclusive results. In a recent RCT [42], infertile women undergoing ICSI, using both fresh and frozen embryo transfers, were randomized to either cholecalciferol (50,000 IU/week) supplementation for six weeks (*n* = 42) or placebo (*n* = 43). Higher clinical pregnancy rates were shown in the vitamin D group compared with the placebo group (38.1% vs. 20.1%, *p* = 0.019) [42]. On the other hand, another RCT including 128 infertile women with vitamin D insufficiency, who underwent frozen-thawed embryo transfer cycles after IVF/ICSI and were treated either with cholecalciferol (50,000 IU/week) for 6-8 weeks (*n* = 57) or with no intervention (*n* = 57) [43], did not show any difference in clinical pregnancy rates between the two groups (25.5% vs. 21.8%, respectively; *p* = 0.810) [43]. Although safe conclusions cannot be drawn by these two studies [of note, both were conducted in Iran, with baseline 25(OH)D concentrations of 12.7–15.8 ng/mL], one should underline some differences between them, which may have had an impact on this discrepancy. First, participants of the former study [42] were of normal body weight in contrast to those of the latter (mean BMI >26 kg/m^2^) [43]. Second, the type of fertilization and the method of embryo transfer (i.e., fresh or frozen) also differed between studies. Third, 25(OH)D concentrations achieved after vitamin D supplementation also differed between studies (37 ng [42] vs. 47 ng/mL [43], respectively).

With respect to PCOS patients, there is evidence for a possible beneficial effect of vitamin D supplementation on fertility outcomes. Particularly, a recent meta-analysis of nine RCTs (502 PCOS women) showed that vitamin D supplementation (in different doses) resulted in increased number of dominant follicles (>14 mm) compared with placebo or metformin (1000–1500 mg/day) (OR: 2.34, 95% CI 1.39 to 3.92, four RCTs) [44]. Moreover, the same meta-analysis showed that vitamin D supplementation especially combined with metformin in women with PCOS seems to regulate menstrual cycles compared with women treated with metformin only (OR 1.85, 95% CI 1.01 to 3.39, three RCTs) [44]. In addition, a recent double-blinded RCT was conducted aiming to evaluate the role of vitamin D supplementation on ICSI outcomes in 105 PCOS infertile women. Patients were randomized either to treatment group (vitamin E, 400 mg/day and vitamin D3, 50,000 IU every two weeks, *n* = 52) or placebo group (*n* = 53) for eight weeks. A higher clinical pregnancy rate was observed in the treatment group compared with the placebo group (62.1% vs. 22.6%; *p* = 0.002), suggesting thus a beneficial effect of combined supplementation of vitamin D and E on ICSI outcomes in PCOS patients [45].

Thus, accumulating evidence supports the notion that vitamin D may play an important role in female fertility. Observational studies associate vitamin D status with IVF outcome, endometriosis and reproductive success after ovulation induction in women with PCOS (Table 1). However, RCTs have not yet established the effect of vitamin D supplementation on the success of ART. Hence, while there is no level 1 evidence, vitamin D sufficiency may be required for improving female fertility outcome. 

## 3. Obesity and Human Infertility

### 3.1. Obesity and Male Infertility (Observational Studies)

Even though sustained interest has recently been shown regarding the role of obesity in male infertility, numerous studies investigating the effect of excessive adiposity on sperm parameters, as well as on reproductive outcomes, have yielded controversial results. A meta-analysis published in 2010 [46] found no association between increased BMI and semen parameters, such as sperm concentration (regression coefficient −0.02; 95% CI −8.24 to +8.18), total sperm count (regression coefficient 12.43; 95% CI −164.95 to +189.81), semen volume (regression coefficient 0.05; 95% CI −0.05 to +0.15) and sperm motility (regression coefficient −1.07; 95% CI −7.39 to +5.25) (five cross-sectional studies). On the other hand, another meta-analysis, published in 2013, which included 21 studies (13,077 participants), showed that both overweight and obese men are at an increased risk of abnormal sperm count compared with men of normal weight [47]. In detail, the risk of oligozoospermia or azoospermia was significantly higher in overweight (OR 1.11, 95% CI 1.01 to 1.21) and obese men (OR 1.28, 95% CI 1.06 to 1.55), compared with those of normal weight. Ιn another systematic review performed in 2015 (30 cross-sectional studies, 115,158 participants), no differences were found regarding semen parameters, such as ejaculate volume (WMD −0.11; 95% CI −0.29 to +0.07), normal morphology (WMD −1.36; 95% CI −4.09 to +1.38) and sperm concentration (WMD −0.53; 95% CI −4.27 to +3.22) between obese men and their counterparts of normal weight. However, it was demonstrated that male obesity negatively affected reproductive potential, as shown by the increased infertility likelihood (OR 1.66, 95% CI 1.53 to 1.79; two studies) and the decreased live birth rates after ART (OR 0.65, 95% CI 0.44 to 0.97; five studies) in the obese men compared with the normal weight men [48]. It seems that the association between obesity and semen parameters remains controversial. Although male obesity is supported to be associated with reduced reproductive potential and sperm DNA damage, the long-term consequences of paternal health on offspring health have not been well-elucidated [16,49].

### 3.2. Obesity and Male Infertility (Interventional Studies)

With respect to the effect of weight loss on male fertility, very few data are available regarding the benefits of weight loss strategies on sperm quality [50]. A pilot cohort study evaluated the effect of a weight loss program, based on healthy diet and daily exercise over a 14-week period, on sperm quality in 43 men with unexplained infertility. Weight loss was associated with an increase in total sperm count (193 million; 95% CI 45 to 341) and morphology (4%; 95% CI 1 to 7) [51]. In addition, a recent prospective study (*n* = 23) evaluated the effect of massive weight loss on sperm quality at six months following bariatric surgery [52]. A positive trend, although not significant, in sperm count and motility was observed, whereas a significant increase in semen volume (difference: +0.6 mL, *p* = 0.04) and viability (difference: +10%, *p* = 0.03) was shown. However, evidence from RCTs regarding the benefits of weight loss strategies on IVF outcomes of infertile men is currently lacking, which does not permit for firm conclusions to be drawn.

### 3.3. Obesity and Female Infertility (Observational Studies)

The effect of obesity on the reproductive process is apparent as early as in adolescence, since obese adolescent girls present with earlier menarche than non-obese girls [53]. In addition, a cohort study including women at the age of 20 years has shown a higher risk of anovulatory infertility in obese women (BMI >30 kg/m^2^) compared with those of normal weight (BMI 20–24.9 kg/m^2^) (RR 1.5; 95% CI 1.3 to 1.9) [54]. Furthermore, in a prospective cohort study including 3029 subfertile ovulatory women, obesity was associated with lower spontaneous pregnancy rates (−4% per kg/m^2^ increase in BMI in women with BMI >29 kg/m^2^) [55].

Moreover, it has been demonstrated that obese women are more likely to respond inadequately to ovarian stimulation after ART compared with non-obese women [56]. Particularly, increased BMI is associated with higher gonadotrophin dosage requirement, fewer retrieved oocytes, lower pregnancy and higher miscarriage rates among women undergoing ART [57,58,59,60]. Gonadotrophin resistance may be induced by an inhibitory effect of elevated leptin or increased clearance of gonadotrophins by the excess of fat tissue [61].

Taking into consideration the negative association between high BMI and infertility, it is not surprising that an increasing percentage of women undergoing ART for pregnancy achievement are obese [14]. A meta-analysis of 33 studies, including 47,967 IVF/ICSI cycles, evaluated the effect of increased BMI on IVF outcome. The authors showed that overweight or obese women demonstrate lower clinical pregnancy (RR 0.87, 95% CI 0.80 to 0.95, 15 studies) and live birth rates (RR 0.80, 95% CI 0.71 to 0.90, five studies), as well as higher miscarriage rates (RR 1.36, 95% CI 1.13 to 1.64, 14 studies) compared with women of normal weight (BMI <25 kg/m^2^) [62]. Furthermore, according to the largest retrospective cohort study analyzing 239,127 fresh autologous IVF cycles, there was a progressive decrease in live birth rates with increasing BMI, from a high of 31.4% in normal BMI cycles (BMI: 18.5–24.9 kg/m^2^) to a low of 21.2% (OR 0.52, 95% CI 0.41 to 0.66) in cycles with the highest BMI (>50 kg/m^2^) [63].

### 3.4. Obesity and Female Infertility (Interventional Studies)

Weight loss is advocated in overweight and obese patients undergoing IVF, as the first-line treatment, in order to improve IVF outcome. Several meta-analyses have examined the effect of weight loss, through various interventions, on subsequent fertility outcomes [50,64,65]. The more recent meta-analysis, including 40 studies (14 RCTs), aimed to determine whether weight loss interventions result in improved reproductive outcomes in overweight and obese women [50]. The dietary interventions used were based on caloric restriction, whereas physical activities were based on aerobic exercise. The meta-analysis showed higher pregnancy rates in women who received a combination of diet and exercise prior to fertility treatment compared with controls (RR 1.59, 95% CI 1.01 to 2.50, six RCTs). However, this effect was not evident in terms of live birth rates (RR 1.54, 95% CI 0.93 to 2.56, five RCTs).

Moreover, a recently published retrospective study including >60,000 fresh and frozen cycles, showed a decreased cumulative live birth rate with increasing BMI and age (linear trend *p* < 0.001). Age had a greater impact than BMI on fertility, suggesting that delaying IVF treatment in order to achieve a lower BMI may be unfavorable for older obese women [66]. Thus, the combination of age and BMI should be seriously taken into consideration when recommending weight loss prior to IVF especially in women of advanced age.

In women of reproductive age, who are unable to achieve adequate weight loss through the combination of diet and exercise, bariatric surgery is considered to effectively treat obesity, showing simultaneously a beneficial effect on infertility. Particularly, a meta-analysis (eight retrospective cohort studies, 589 infertile obese women), evaluating the incidence of pregnancy achievement after bariatric surgery in infertile women, showed a weighted mean incidence of spontaneous pregnancy achievement of 58% (95% CI 54 to 62%), after bariatric surgery [67]. However, the available evidence is not robust regarding the efficacy of bariatric surgery on ART outcome in obese infertile women. A retrospective study aiming to evaluate the ART outcomes in 40 infertile obese women before and after bariatric surgery, showed an increase in the number of retrieved oocytes (6.6 ± 1.7 vs. 8.1 ± 2.5, *p* = 0.004), MII oocytes (5.5 ± 1.6 vs. 6.9 ± 2.8, *p* = 0.008) and fertilized oocytes (4.2 ± 1.7 vs. 5.3 ± 2.4, *p* = 0.02). Moreover, pregnancy rates increased by 37.5% (*p* < 0.001) and live birth rates by 35% (*p* < 0.001) following bariatric surgery [68].

**Figure 1:**
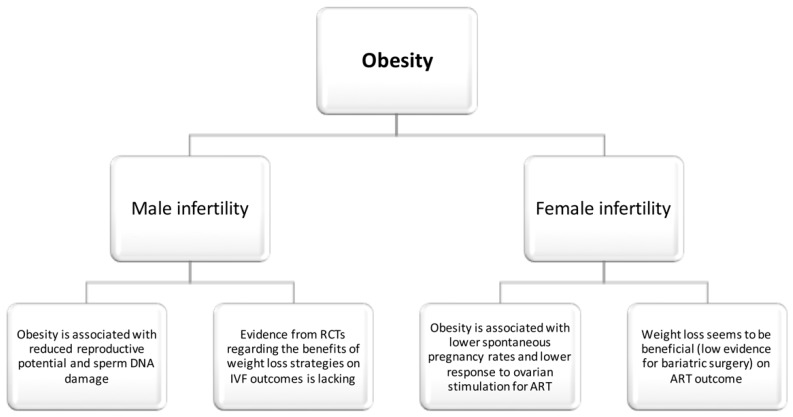
Obesity and infertility.

The aforementioned data suggest a potential detrimental effect of obesity on both male and female fertility, summarized in Figure 1. Weight loss seems to be beneficial, although data from RTCs are needed to adequately estimate the effect of different weight-loss strategies (such as bariatric surgery) on ART outcome.

## 4. Potential Interactions between Vitamin D and Obesity

Despite the evidence of a detrimental effect of diet and obesity on human fertility, it is not clear whether these two states also act in synergy. It has been well-established that obesity is associated with vitamin D deficiency, providing a potential pathogenetic linkage with infertility [69]. Whether it is vitamin D deficiency that contributes to obesity or the contrary, is a matter of debate. Although the underlying mechanisms are not clear, it has been suggested that obesity may lead to low vitamin D concentrations, perhaps due to its accumulation in adipose tissue, leaving only small quantities available in the circulation [70]. Additionally, it has been proposed that obesity can cause vitamin D deficiency through inadequate intake or through the reduced exposure of obese people outside to UV radiation [71]. Furthermore, the enzymes that catalyze the hydroxylation of vitamin D to its active forms are produced in lower concentrations in obese when compared with non-obese patients [72].

A prospective study attempted to assess the association between 25(OH)D concentrations in follicular fluid with body weight, in 199 infertile patients undergoing ICSI. Patients with vitamin D deficiency had significantly higher mean body weight (64.1 kg vs. 60.7 kg, *p* < 0.01) than patients with vitamin D sufficiency. However, no data were provided regarding the reproductive outcome [73], highlighting, thus, the need for further studies to investigate the impact of 25(OH)D concentrations in follicular fluid with respect to reproductive outcome. Moreover, an association between vitamin D concentrations and metabolic disturbances in PCOS, including insulin resistance and other features of the metabolic syndrome, has also been suggested [10]. In addition, obesity also appears to be associated with the severity of PCOS. A prospective study including 100 women with PCOS, evaluated the correlation between serum 25(OH)D concentrations and metabolic parameters in 57 obese and 43 non-obese women with PCOS. It showed that mean 25(OH)D concentrations were significantly lower in the obese compared with the non-obese PCOS patients [12.79 ± 3.76 ng/mL vs. 29.27 ± 8.10 ng/mL, *p* < 0.01] [74]. Furthermore, a comparative study, including 40 PCOS and 40 non-PCOS patients undergoing IVF, assessed the effect of BMI on follicular fluid 25(OH)D concentrations, showing lower concentrations in PCOS patients compared with non-PCOS [4.5 ± 1.7 vs. 7.1 ± 1.3 ng/mL, *p* < 0.05 (normal weight patients); 1.6 ± 0.9 vs. 5.2 ± 1.8 ng/mL, *p* < 0.05, (overweight patients)]. Follicular fluid 25(OH)D concentrations were also negatively correlated with BMI (r = −0.51, *p* < 0.01) [75].

Moreover, it has been shown that VDRs are implicated in the regulation of genes involved in glucose and lipid metabolism, suggesting a potential role of vitamin D deficiency in the pathogenesis of metabolic syndrome [76]. This is supported by the expression of VDR on pancreatic β-cells and the association between VDR gene polymorphisms and insulin resistance [77]. Moreover, a comparative study evaluating VDR expression in granulosa cells showed a strong negative correlation with BMI (r = −0.43, *p* < 0.05) and a significantly lower gene expression of VDR in the PCOS/overweight patients compared with the non-PCOS/normal weight patients (*p* < 0.01) [75].

## 5. Conclusions

Vitamin D deficiency seems to independently affect male and female fertility. Most evidence for this association emerges from experimental and observational human studies. However, robust evidence from interventional studies is currently lacking, since data derive from studies of small sample size and with significant heterogeneity regarding the optimal vitamin D dosage and duration. Furthermore, a more beneficial effect in severe vitamin D infertile subjects cannot be excluded. On the other hand, obesity also seems to negatively affect fertility in both genders. Weight loss, especially after bariatric surgery, may exert a beneficial effect on fertility outcomes, since it may increase both the number of retrieved and fertilized oocytes, top-quality embryos, pregnancy and live birth rates. The exact pathogenetic mechanisms linking vitamin D with obesity cannot be fully elucidated and, thus, a rather synergistic effect in the field of infertility may be supported. In any case, well-designed interventional studies, with live birth rates as the primary outcome both in vitamin D deficiency and obesity states, are needed.

## Figures and Tables

**Table 1:** Vitamin D and infertility.

**Male infertility** Linear or U-shaped correlation between vitamin D concentrations and sperm motility/morphology [23,24,25,26,27,28]. Sufficient vitamin D concentrations associated with high testosterone concentrations [29,30,31]. Supplementation of vitamin D improved semen quality and pregnancy rates [32,33]. Supplementation of vitamin D increased testosterone concentrations [34,35].
**Female infertility** Contradictory data on whether supplementation of vitamin D is associated with pregnancy rates [42,43].
**ART** Higher live birth rates in vitamin D-sufficient women [37,38].
**Endometriosis** Linear correlation between vitamin D concentrations and diameter of ovarian endometriomas [39]. Higher incidence of vitamin D deficiency/insufficiency in women with endometriosis [40].
**PCOS** Linear correlation between vitamin D levels and reproductive success rates after ovulation induction in women with PCOS [41].

**Abbreviations**: ART: assisted reproductive techniques; PCOS: polycystic ovary syndrome.
